# Potential Impacts of Climate Change on the Toxicity of Pesticides towards Earthworms

**DOI:** 10.1155/2021/8527991

**Published:** 2021-08-20

**Authors:** H. Kaka, P. A. Opute, M. S. Maboeta

**Affiliations:** ^1^Unit for Environmental Sciences and Management, North-West University, Private Bag X6001, Potchefstroom 2520, South Africa; ^2^Department of Animal and Environmental Biology, Faculty of Life Sciences, University of Benin, Benin City, Nigeria

## Abstract

This review examined one of the effects of climate change that has only recently received attention, i.e., climate change impacts on the distribution and toxicity of chemical contaminants in the environment. As ecosystem engineers, earthworms are potentially threatened by the increasing use of pesticides. Increases in temperature, precipitation regime changes, and related extreme climate events can potentially affect pesticide toxicity. This review of original research articles, reviews, and governmental and intergovernmental reports focused on the interactions between toxicants and environmental parameters. The latter included temperature, moisture, acidification, hypoxia, soil carbon cycle, and soil dynamics, as altered by climate change. Dynamic interactions between climate change and contaminants can be particularly problematic for organisms since organisms have an upper and lower physiological range, resulting in impacts on their acclimatization capacity. Climate change variables such as temperature and soil moisture also have an impact on acidification. An increase in temperature will impact precipitation which might impact soil pH. Also, an increase in precipitation can result in flooding which can reduce the population of earthworms by not giving juvenile earthworms enough time to develop into reproductive adults. As an independent stressor, hypoxia can affect soil organisms, alter bioavailability, and increase the toxicity of chemicals in some cases. Climate change variables, especially temperature and soil moisture, significantly affect the bioavailability of pesticides in the soil and the growth and reproduction of earthworm species.

## 1. Introduction

Climate change is defined as the alteration in the climate state, evidenced by changes in the variability of its properties such as temperature, precipitation, and wind [1]. These variables persist for an extended period (typically decades or longer) which coincides with an increased likelihood in the intensity of extreme climate events, such as drought and flooding [2, 3]. Soil biodiversity includes many different types of organisms ranging from microscopic organisms to large animals and plants. The soil supports a wide range of ecosystem functions, processes, and services, making it essential to human life [4–7]. At some point, environmental processes in the air, water, and soil interact with each other, and this interaction is essential for these processes to function at an optimal level. Anthropogenic activities including agricultural and mining activities are primarily responsible for soil pollution [8–10] which impacts the biodiversity of soil organisms which are therefore reduced or lost [11].

Earthworms make up 40–90% of the soil macrofaunal biomass in many terrestrial ecosystems and thus are vital soil organisms [3]. There are 23 families and over 700 genera with more than 7,000 species of earthworms already described worldwide, although the number of species is much more [3, 12]. They play an important role as ecosystem engineers, thus making them keystone species [3]. The environment is modified physically and chemically by earthworms; they create, transform, and maintain the habitat for soil organisms and plant communities [5]. These modifications are done by processes such as litter fragmentation, burrowing, and casting activities. These activities drive soil processes such as nutrient cycling, soil aggregate stability, water infiltration, plant growth, and soil carbon storage [13]. They have many roles in the ecosystem, making them very useful as bioindicators in soil ecotoxicological studies [14–16]. The diversity of earthworm species is studied worldwide, and Phillips et al. [17] highlighted climate change drivers such as increasing air temperature and changing mean annual rainfall as key factors affecting its global distribution. Climate change alters soil properties such as moisture, temperature, pH, and texture, affecting not only earthworm communities but also all life interacting with the soil environment [3].

## 2. Predicted Global Change Scenarios

Climate change is a global problem. Generally, when models are used to predict future changes in climate, it usually suggests an increase in more extreme and severe events in the coming decades [1]. The main drivers of climate change affecting the soil ecosystem are greenhouse gases, air and soil temperature increases, extreme precipitation, and erosion [18, 19]. According to the Intergovernmental Panel on Climate Change [20], global warming for 2006–2015 was 0.87°C, which is higher than the average between 1850 and 1900. The estimated global warming caused by anthropogenic activity is currently increasing at 0.2°C per decade due to the past and ongoing impact of pollutants [20]. Activities by humans have caused global warming to increase by 1°C above preindustrial levels, and this is likely to reach 1.5°C between 2030 and 2052 if it continues to increase at this current rate [20]. When comparing regional changes of the preindustrial levels to the 1.5°C increase in global warming, it estimates extreme increased temperatures, increased heavy precipitation, and an increase in drought in many regions, for example, the projected increase in precipitation in West Africa, the increase in temperature in the Indian Ocean resulting in increased rain in East Africa, and the increased global surface temperature [20]. These predicted climate change events may have significant effects on the bioavailability of pollutants in the soil.

**Figure 1:**
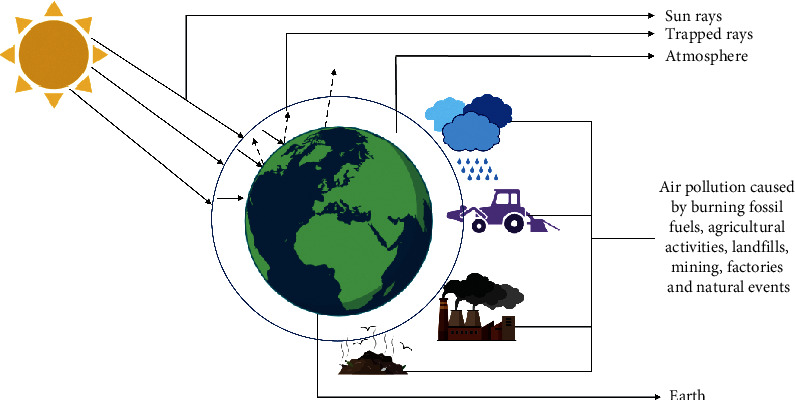
Simple representation of the greenhouse effect. The main drivers of air pollution: burning of fossil fuels and industrial and agricultural activities. These events trap more of the sun's radiation in the atmosphere resulting in global warming.

The ability of greenhouse gases such as carbon dioxide, methane, nitrous oxide, and fluorinated gases to capture heat from the sun's energy causes the greenhouse effect [21]. The primary sources of air pollution are the combustion of fossil fuels, as well as industrial and agricultural activities. As a result of these events, more of the sun's radiation is trapped in the atmosphere, causing global warming (Figure 1). According to Olivier and Peters [22], since the year 2012, the global annual increase in CO_2_ emissions has slowed down to about 1.5%, and in 2015-2016, it has remained flat. In 2017, 2018, and 2019, the global CO_2_ emissions increased by 1.4, 2.4, and 0.9%, respectively. The increase was a result of increased consumption in coal. The annual global carbon sequestration potential is about 0.4–0.7 Pg C y^−1^, which means that if these rates remain constant until the year 2100, the soil carbon sequestration would only contribute to a maximum of 1–3% towards reducing the carbon emissions [23]. This scenario suggests that carbon sequestration into the soil will play a minimal role in reducing carbon dioxide emissions. The study conducted by Marhan et al. [24] indicated a higher loss of nitrogen in the form of N_2_O from the soil with high earthworm population and a warmer climate.

**Figure 2:**
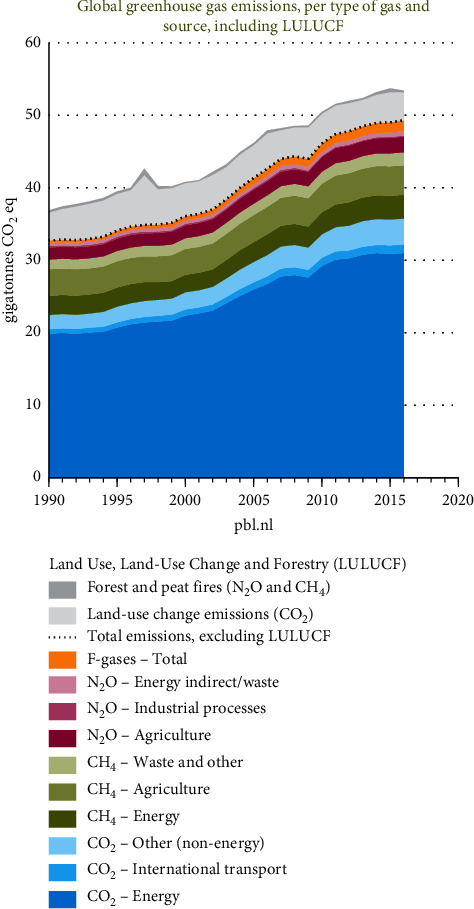
Global greenhouse gas emissions, per type of gas and source, including LULUCF (source with permission: Olivier and Peters [22], Trends in global CO_2_ and total greenhouse gas emissions: 2019 report, PBL Netherlands Environmental Assessment Agency, The Hague).

As shown in Figure 2, greenhouse gases are increasing at an alarming rate, and the increase has been exponential over the last ±60 years. If left unchecked, this extreme increase will keep on having a massive effect on environments worldwide. Human activity is responsible for the rapid rise in greenhouse gases such as carbon dioxide, methane, and nitrous oxide [25]. Most of the gas emitted is carbon dioxide, although gases such as methane and nitrous oxide are much better at absorbing infrared radiation and contributing to global warming [25]. Over 100 years, 1 kg of methane has a warming potential 23 times greater than 1 kg of carbon dioxide, and 1 kg of nitrous oxide has a warming potential of nearly 300 times that of carbon dioxide [25]. Approximately two-thirds of the nitrous oxide emissions and one-third of the atmosphere's methane emissions come from soils [25]. If global warming continues unabated, it can have many devastating effects such as rising sea levels, ocean acidification, and severe weather events. However, there have been international initiatives such as the Kyoto Protocol, the signing of the Paris Agreement on climate change, and other initiatives set up to combat this effect globally [21]. According to Olivier et al. [26], the record for the warmest year in 140 years was 2016 with an increase of +0.99°C, and in 2019, it was the second warmest year with the global land and ocean surface temperatures increasing by +0.95°C above the average. They further stated that, since 1880, 9 out of the 10 warmest years have occurred since 2005.

Anthropogenic activity has had an influence on the global water cycle and in turn affected the global precipitation regime by globally increasing the amount of atmospheric moisture and the precipitation patterns over the land [1]. Singh et al. [3] stated that precipitation regime changes might show significant heterogeneity and thus be difficult to predict. However, climate models project an increase in the frequency of extreme precipitation events. Also, an increase in rainfall due to climate change can result in soil loss and soil erosion by increasing sedimentation in streams and reservoirs [19]. This soil loss will have detrimental effects on soil organisms as well as soil functions in an ecosystem. Once these functions are disrupted, changes in the environment can occur. Most of water which supplies soil moisture comes in the form of precipitation and is evaporated from the ocean and transported to the land by the atmosphere, and this movement of water in a climate system is an essential part of life on land.

Ultraviolet radiation is one of the components of solar radiation, and it is separated into UV-A, UV-B, and UV-C [27]. Stratospheric ozone usually reflects UV-C and UV-B; thus, only UV-A and very little UV-B reach the Earth, although due to the depletion of atmospheric ozone, there is an increase in UV-B reaching the Earth [27, 28]. Higher UV-B radiation modifies soil microbial communities and decreases populations of soil meso- and macrofauna [28]. According to Formánek et al. [28], UV-B is known to increase degradation of some pollutants such as phenylurea herbicides, p,p'-DDT, 2,4-dichlorophenoxyacetic acid, biphenol, and PAHs, although it can also inhibit rhizoremediation of organic pollutants. In arid and semiarid regions, ultraviolet radiation is one of the main drivers of increasing litter decay through photodegradation [29]. Abiotic photodegradation can mineralize the decomposition of litter into gases such as CO_2_, CO, and CH_4_ and, at the same time, alter the chemistry of organic materials [29]. Complex carbon structures can be broken down by ultraviolet radiation into smaller molecular compounds, increasing dissolved organic carbon concentration in the litter [29].

## 3. Consequences of Climate Change for the Structure and Function of Soil Ecosystems

According to Bellard et al. [30], in fifty years from now, we are likely to see an increase in the rate of species loss due to climate change, although the evidence that this will happen is still inconclusive. The aboveground and belowground ecosystems have many interactions, and they rely on one another for specific functions [31]. However, the aboveground biodiversity is usually observed and acts as a basis for predicting environmental changes [32]. The need to study the biodiversity of aboveground and belowground ecosystems simultaneously is essential because we might not find the differences found in one ecosystem in the other [33]. Anthropogenic activities contribute to the loss of biodiversity and ecosystem services in soil. Mining and agriculture, for example, exploit the soil ecosystem in an unsustainable manner, potentially resulting in a 60% depletion of ecosystem services [7, 34]. Soils and soil processes will primarily be affected by climate change through changes in rainfall patterns and temperature. Earthworm populations will be affected by climate change variables such as drought, irregular precipitation, and increasing temperatures [35–37]. Additional research is required on climate change effects on soil organisms to develop and improve models [38]. Irrespective of the fact that earthworms and other soil organisms play a vital role in soil ecosystems and functions and the noticeable danger of climate change, there is still no conclusive overview of climate change effects on soil organisms, particularly earthworms, given the increasing pesticide pollution in the environment.

### 3.1. Rise in Soil Temperature

Temperature is one of the most important climate change drivers, and it is influential in determining soil biological activity and the decomposition process [3]. The increase in temperature has shown to be the cause of various alterations to many plant and animal species, with it being massively influenced by biotic interactions [39, 40]. Earthworms are a poikilothermic species, which means that their body temperatures fluctuate with their respective environments; therefore, the activity, density, growth, metabolism, respiration, and reproduction of earthworms are all affected by temperature variations. They tend to gather in areas where conditions are ideal for their metabolism, suggesting that both high and low temperatures produce a direct response; these effects include reduced growth rate, feeding activity, cocoon production, and juvenile development both at the individual and community level [3]. Although more is known about the higher lethal temperatures than the lower lethal temperatures, the response of earthworms to temperature fluctuations is also dependent on the species involved because every species has its specific tolerance range for different variables. The increase in temperature can also affect the bioavailability of the soil's metal pollutants. Besides the fact that temperature will influence soil organisms in the natural world, many other variables, such as the soil moisture content, will also affect the ecosystem at the same time.

A study conducted by Eggleton et al. [41] showed earthworm diversity over six years and took into account temperature and soil moisture as climate change variables. They reported a decrease in earthworm numbers in the dry winter months, and during the moist summer months, there was an increase in earthworm numbers; dry summer months negatively affected epigeic species such as *Dendrobaena octaedra*, *Dendrobaena attemsi*, and *Satchellius mammalis*, and in wetter months, the earthworm numbers increased. Species such as *Aporrectodea caliginosa* and *Lumbricus rubellus* had a high tolerance to temperature changes. Also, the study conducted by Berman and Meshcheryakova [42] indicated a similar result: in higher temperatures with higher soil moisture, earthworm numbers increased rapidly. The study conducted by Hughes et al. [43] showed that higher air temperatures with lower soil moisture could reduce the earthworm population. The abundance of earthworms usually increases when climatic conditions are favourable, such as when the temperature is moderate and the soil moisture content is higher [17]. This abundance tends to decrease in colder climates, where the soil moisture content is lower [44].

### 3.2. Change in the Soil Moisture Content

The soil environment is affected directly and indirectly by drought [45]. The deficiency of precipitation or drought does not immediately affect the deeper soil layers but will reduce the water content of the upper soil layer relatively quickly [46]. The movement of soil organisms is inhibited by reduced soil water content, which hardens the soil [47] and diminishes the extent of the water film [13]. The reduction of vegetation cover by drought can cause increased temperatures, an altered microclimate on the soil surface, and reduced resource availability [48]. The biological activity and earthworm diversity of soils are reduced through increased soil temperature both directly and indirectly. The frequency and significance of droughts are increasing around the world [49], and there are only a few studies on the effects of drought on earthworms [50–54]. An increase in temperature is associated with the volatilization and degradation of organic and inorganic pollutants in the soil [18]. Thus, an increase in soil temperature increases contaminants' transportation in the ground [18].

Earthworms are only active if free water is available in the soil. They are morphologically and physiologically limited in their use of the cuticle to maintain body moisture [3]. When soils become too dry, earthworms usually lose weight, decrease their burrowing activity, and may also enter diapause or become dormant [52]. The cocoons produced by epigeic species are drought resistant although it represents an essential strategy for the survival of the species in drought-stricken areas [53] and may rapidly recover after the drought conditions end [41]. Anecic earthworms can form permanent vertical burrows in the soil, during the dry periods, can enter a diapause, and stay dormant for several months [3]. Some endogeic species such as *Aporrectodea caliginosa* can form nonpermanent horizontal burrows in the topsoil and form aestivation chambers covered with mucus and gut content and protect themselves against water loss [3]. The composition of earthworm community's response to droughts can likely change due to different ecological strategies these species use. Plum and Filser [54] suggested that soil's organic matter and water-holding capacity were critical factors in modifying earthworms' responses to drought conditions.

Floods, on the contrary, can cause massive changes to the soil [55] by oversaturation, and this can result in a lack of oxygen in the soil resulting in hypoxia, although the floodplains contain sediment which is very nutrient-rich making them some of the most productive ecosystems around the world [56]. The diffusion and oxygen availability of the soil are reduced during flooding, resulting in reduced soil nutrient availability since decomposition processes are halted [3]. Anaerobic conditions occur in flooded soils, which will significantly affect the composition of soil food webs, microbial biomass, and soil microbial community structure [57, 58]. Plum [57] stated that flooding reduced the abundance, biomass, and diversity of all groups of soil organisms in grasslands, and an increase in temperature and flood duration increased its effects. The ability of soil invertebrates to survive depends on their behavioural, morphological, and physiological traits [57]. Singh et al. [3] reported that the abundance of earthworms was reduced in floodplain areas and that periodical flooding had species-specific effects on earthworm populations. Another study conducted by Bullinger-Weber et al. [59] also indicated that the absence of anecic earthworms would suggest that more erosion and sedimentation processes have taken place, and an increase in epigeic earthworms may have positive effects on the texture of the topsoil and the organic matter quality. Many earthworms can survive long periods submerged in water [3], and some earthworm species can survive in flooded soils [60]. A critical factor to consider is the period between two flooding events. The study conducted by Klok et al. [61] suggested that earthworms from a frequently flooded area mature much quicker than earthworms from a nonflooding site. Floods can also reduce earthworm populations by not giving juvenile earthworms enough time to develop into reproductive adults. Short flooding periods followed by long recovery periods can be beneficial to certain earthworm species, e.g., *Lumbricus rubellus* and *Allolobophora chlorotica*. Schütz et al. [62] and Bullinger-Weber et al. [59] suggested that they could favour epigeic earthworm species. A study conducted by Plum and Filser [54] indicated that controlled flooding should be kept short in winters and natural summer flooding should follow and that a recovery period of six months should be sufficient for the reestablishment of earthworm populations.

### 3.3. Soil Acidification

The soil acidification process is a gradual and continuous natural process that occurs during pedogenesis and is aided by water leaching basic cations to lower subsoil [63]. The acidification is accelerated by an increase in nitrogen and sulphur deposition which is associated with human activity [64]. Soil pH has a fundamental effect on the solubility and availability of potentially toxic ions, and while low pH (acidic) benefits free metal cations and protonated anions, higher pH (alkaline) will favour carbonates and hydroxyl complexes [63]. Acidification can be enhanced by high precipitation which will allow leaching of soil cations. However, the soil itself is a buffer for external H ions [64], and only once it exceeds its maximum capacity will the soil become acidic.

One of the main drivers of soil acidification in terrestrial environments is the accumulated nitrogen deposition, and according to Tian and Niu [65], during 2000–2010, more than 50 kg·ha^−1^ of nitrogen was accumulated in terrestrial environments. This nitrogen-induced acidification has posed a significant threat to terrestrial ecosystem functioning and species diversity. Using nitrogen fertilizers can have a variety of effects on soil acidification. The NH_4_^+^ ions can displace base cations such as Ca^2+^, Mg^2+^, and Na^+^ by binding them and making them more easily leached out of the soils, thus reducing their buffering against acidification, and therefore, when plant roots absorb the NH_4_^+^ ions, the H^+^ ion is released into the soil solution, and this can cause soil acidification [65]. Climate change variables such as temperature and soil moisture affect the acidification process. Higher soil moisture will allow leaching to occur more easily promoting soil acidification. Reducing the nitrogen cycles by lowered temperatures will reduce the ecosystems' ability to sequester nitrogen resulting in more acidification [63]. When precipitation is higher, it accelerates the leaching of cations, further aggravating acidification [64].

### 3.4. Soil Organic Carbon (SOC) Cycling and SOC Dynamics

The carbon cycle involves the soil, plants, and all animal life, including humans; thus, the disruption of the carbon cycle would mean disaster for all living organisms. Soil organic matter is expressed as soil organic carbon, which plays a vital role in the sorption of soil pollutants [18]. The basic processes involved in the global carbon cycle start when plants take up carbon dioxide from the atmosphere. Through photosynthesis, sunlight energy is trapped in the carbon-to-carbon bonds of organic molecules. Some of these organic molecules are used as energy sources, while carbon is returned to the atmosphere as carbon dioxide. The remaining organic materials are temporarily stored as constituents of the standing vegetation, which eventually add to the soil as plant litter or root deposition.

The productivity of the vegetation growing on the soil is related to the soil's carbon input rate, measured by the net primary production (NPP) [23]. The NPP varies with climate, land cover, species composition, and soil type and also shows seasonal variations due to its dependence on light and temperature. Smith [23] explained that, over long periods, an amount of NPP enters the soil as organic matter (decomposition of plant matter) and is converted into carbon dioxide and methane via soil heterotrophic respiration processes. Remaining carbon is referred to as net ecosystem production (NEP). Although harvesting, fires, and insect damage can also remove carbon, when combined with heterotrophic processes, they can counterbalance the terrestrial carbon dioxide input from global primary production, and residual carbon is known as net biome production (NBP), which can be positive or negative depending on whether the ecosystem is a carbon source or sink [23].

Human impact disrupts the carbon cycle balance because carbon enters the atmosphere from burning of fossil, cement production, the ocean, land use, animals, plants, and geological reservoirs through mining. The emission rate of carbon in the atmosphere remains unbalanced, and the speed at which carbon is used makes it nonrenewable, which disrupts the global carbon cycle. Climate, vegetation, parent material, topography, and time are all important factors influencing soil carbon. Soil carbon reservoir has been suggested as both a sink and a source of atmospheric carbon dioxide. It is considered a source when net decomposition exceeds carbon inputs into the soil, which could be due to human activities or global warming, and it is considered a sink when the difference between net ecosystem carbon uptake and tree growth rates exceeds the net ecosystem carbon uptake [66]. The degree and timing of response are determined by the amount of carbon available to respond rapidly to climate and vegetation changes, as well as the time lag between carbon fixation in plants and subsequent release to the atmosphere during decomposition.

## 4. Toxicity of Pesticides towards Earthworms under Climate Change Scenarios

### 4.1. The Fate of Pesticides

Climate change has left indelible imprints on the ecosystem, including extreme temperature and rainfall events, as well as an increase in atmospheric carbon dioxide (CO_2_) concentrations, which may significantly affect the usage, distribution, and degradation patterns of pollutants such as pesticides [67]. Weather variables such as rainfall, temperature, and wind extremes influence pesticide fate and behaviour in the environment. It is essential to understand the environmental fate of pesticides because they are biologically active and designed to interfere with metabolic processes. The potential health risks that pesticides can have on human and environmental health need to be evaluated, after which one must take precautions to mitigate their effects. Applying pesticides to targeted areas predictably allows these chemicals to affect organisms and the environment. It is in the understanding of pesticides' physical, chemical, and biological processes that one can realize their impact on target and nontarget species. This understanding is crucial for improving pest management strategies that must have minimal adverse effects on human and environmental health. One of the most critical factors in the transport of pesticides in the soil is the sorption-desorption process because it controls the volume of pesticides available for distribution [68].

In terms of pesticide efficacy, it was widely assumed that some pesticides are more hazardous to insect pests at higher temperatures [69]. However, recent reports such as [70] discovered that chlorpyrifos decomposed more at higher temperatures, resulting in decreased mortality and oxidative damage to insect pests. This is attributable to the fact that organophosphate insecticides hydrolyze at a faster rate at higher temperatures [71]. Pesticides that undergo aqueous-phase hydrolysis, such as organophosphates, carbamates, synthetic pyrethroids, and sulfonylureas, are generally temperature-sensitive [72]. When exposed to intense sunlight, some insecticides undergo substantial photodegradation. The loss of pesticides due to volatilization is also favoured by rising temperatures [73]. Microbial activity in soil will also increase when temperature and moisture levels rise. As a result, the rate of pesticide degradation induced by soil microbes will increase. Thus, with the higher dissipation of pesticides and the climate adaptation of pests as a result of global warming, pesticides will most likely be used more frequently and at a higher application rate [72]. The uncertainties related to climate predictions make it challenging to predict the effect of climate change on pesticides. However, according to Bloomfield et al. [74], changes in temperature and seasonality and intensity of rainfall are the main drivers in pesticide fate and transport. Also, changes in land use accompanied by climate change events will substantially affect the fate of pesticides in the soil environment.

**Figure 3:**
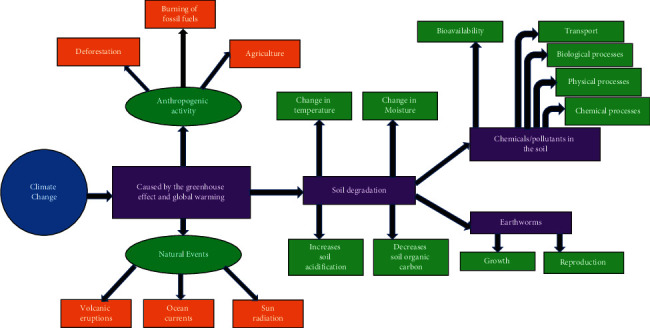
Fate and behaviour of pesticides towards earthworms under the influence of climate change.

Figure 3 illustrates a simple representation of how climate change affects soil pesticides and inevitably soil organisms, specifically earthworms. Climate change is mainly caused by the greenhouse effect which is a natural effect that is accelerated by anthropogenic activities such as deforestation, burning of fossil fuels, and agricultural practices. Deforestation and agriculture can go hand in hand because according to Bennett [75], 25% of the world's greenhouse gas emissions comes from deforestation through practices such as logging and burning of biomass, thus making agriculture one of the most important causes of deforestation. As stated above, climate change does have a great effect on the soil ecosystem, and as a result, it will influence soil organisms as well as chemicals in the ground. Several studies have found that climate change affects soil dynamics, which in turn affects pesticide toxicity to earthworms (Table 1).

### 4.2. Effect of Climate Change Drivers on Pesticide Toxicity to Earthworms

Chemical pollutants including pesticides can be altered by climate change when the climate change drivers alter the physical, chemical, and biological properties of ecosystems [85]. The impacts of climate change on pesticides may be both indirect and direct [86]. The indirect effects include changes in pesticide exposure due to the shifts in cultivation towards higher latitudes and extension of cultivation periods [74, 87]. Potential enhancement of pesticide volatility and degradation could affect the effectiveness of pesticides against pests and thus could increase application levels for pesticides [88]. This rise in the use of pesticides may be in both quantity and scope of application [89]. In terms of direct effects, climate change can affect the decomposition and toxicity of the pesticides [86], especially in the expected temperature and precipitation alternations [88]. Degradation of the pesticide is dependent on soil moisture and temperature [90]. Soil temperature and soil moisture are key climate change drivers influencing earthworm growth, survival, fecundity, and behaviour [91] and indirectly influencing the environment of the earthworm and food availability [92]. Also, soil temperature and moisture influence most characteristics of the life cycle, such as weight, cocoon incubation time, sexual maturity initiation, reproduction, and life span. Temperature rise can speed earthworm growth and reproduction rate [78]. It has also been shown that, in *Aporrectodea caliginosa* earthworms, soil moisture and temperature affect biomarkers [77]. Synergistic responses have been reported in many studies dealing with the impact of soil moisture on effects of various chemicals [76, 93–95].

A study done by González-Alcaraz et al. [96] assessed how different combinations of air temperatures and soil moisture contents (20°C and 25°C; 30% and 50%) affected the bioaccumulation kinetics of zinc and cadmium in the earthworm species *Eisenia andrei*. The results of this study indicated that the earthworms accumulated zinc rapidly in contaminated soils. Still, when put in uncontaminated soil, they quickly eliminated zinc. The air temperatures of 20 and 25°C and the soil moisture contents of 30% and 50% had no significant impacts on zinc bioaccumulation in the earthworms. However, higher temperatures and moisture allowed cadmium to be taken up and eliminated at faster rates. The kinetics of cadmium reduced when the temperatures were higher, coupled with lower moisture contents. Analysis already performed in other studies indicates the value of ecotoxicological studies and the fact that climate change variables influence the bioavailability of pollutants in variables of soil climate change must be considered while performing these experiments. The study on *Eisenia fetida* species by Pelosi et al. [97] also reported how climate change can affect soil organisms as well as contaminants in the soil. According to their findings, temperature and soil moisture content influenced soil enzyme behaviour as earthworms responded to pesticides differently. The toxicity of earthworms varied when exposed to the same pesticide concentration at different temperatures, decreasing at lower temperatures and increasing at higher temperatures. This report shows that temperature variability can modify the effects of pesticides on earthworm populations. However, more research is required to better understand the complex relationship between environmental factors and the toxicity of chemicals, especially the dynamic interaction between climate change drivers and pesticide toxicity to earthworms. Toxicokinetics, bioaccumulation, and detailed life-cycle traits in earthworm toxicology research can help explain the exposure pathways taken by soil pollutants in tested species under current projected climate change scenarios which will help in better understanding the effects of these pollutants in soil and soil vertebrates with the aim of taking comprehensive mitigation strategies as well as sustainable use of agricultural pesticides.

## 5. Discussion and Conclusions

The soil ecosystem is a critical environment that provides many services. It stores carbon, provides a medium for plant growth, and serves as a natural environment for organisms. The soil environment interacts with the atmosphere and hydrosphere and provides many human civilisation materials and food to consume. Thus, the soil environment's health is crucial for human, plant, and animal life, and protecting this natural resource is essential for survival. Activities such as mining and agriculture provide a source of pollution to the soil environment. Mining disrupts the Earth's crust and displaces large volumes of soil, ruining the environment. Refining mined materials pollutes the soil and water systems. Also, large-scale agricultural practices alter the natural ecosystem, and the use of pesticides can pollute the soil and water systems through runoff and leaching. Earthworms are keystone species and are generally used in ecotoxicological studies since they interact with the soil directly and perform many functions in soil processes [15, 98–100]. The interaction between earthworms and soil makes them a relevant species to be used in laboratory tests. There are many different earthworm species used depending on the region and tests done. Most soil ecotoxicological studies use earthworms in their tests, [8, 15, 16, 80, 96, 98, 99] to name a few, and this gives further evidence that earthworms are a suitable species to use in these studies. Table 1 shows the results of studies on the effect of pesticides towards earthworms under climate change scenarios. Different concentrations of pesticides had different effects on various life-cycle traits of earthworms and biomarkers. When climate change variables are introduced, these same concentrations had different effects on the same tested parameters, and this will again supply more evidence for researchers to take these variables into account.

Global climate change has an impact on ecosystems worldwide. The focus of this review is on the potential effects of climate change on pesticides and their implications for earthworms in the soil ecosystem. Climate change has been shown to have a significant impact on the toxicity of pollutants in soil by altering weather patterns and natural global cycles. Previous research has found that varying temperature and moisture affect the bioavailability of pollutants in the soil ecosystem. According to many analyses from the Intergovernmental Panel on Climate Change (IPCC) report, the global temperature and other climate change drivers are becoming more severe and erratic; therefore, current research must consider environmental concerns related to climate change. Year 2019 was the second warmest year in 140 years with an increase of 0.95°C [22], and the reality is that global warming is affecting the human and natural world. This review highlights some of the impacts of climate change on the soil and the pollutants in the soil. Most toxicological analyses using earthworms do not take these climate change variables into account; thus, they use standard temperatures and soil moisture contents while focusing mainly on single pollutants and their effects. However, these variables undoubtedly affect the bioavailability of these pollutants in the soil. To accurately determine the effects of these pollutants in natural ecosystems, one must consider these variables. Studies such as [22, 39, 40, 85, 101], to name a few, all indicate that climate change can directly affect the soil temperature and moisture. When air temperatures increase, the temperature of the soil surface increases as well, and an increase in temperature will also reduce the soil moisture. An increase in the temperature and a decrease in the soil moisture will affect the characteristics as well as the bioavailability of pollutants in the soil. Climate change also makes rainfall patterns more unpredictable, and this could lead to flooding and droughts. Both events can cause the soil moisture to become excess (flooding) or minimal (droughts). Excess water can cause leaching in the soil which will promote acidification and runoff from agricultural areas and mines which can pollute nearby areas. Droughts can reduce the vegetation cover resulting in increased soil erosion. These conditions will also affect earthworm populations as well as the bioavailability of contaminants in the soil.

Future studies must consider collecting relevant data as climate change becomes a more urgent issue. Several studies such as [68, 85, 96, 101–103] all show that other factors such as temperature and soil moisture affect the bioavailability of pesticides in the soil, thus making it essential to be taken into account and not only using the concentrations of pollutants as an endpoint.

The soil itself has capabilities to act as a buffer against pollutants as well as variables such as temperature, moisture, and acidification. The properties and make-up of the soil give it these characteristics to act as a buffer. The organic matter (carbon) in the soil can help reduce the effects of heavy metals, for instance, in the soil. The variety of microbial communities in the soil also helps to break down organic pollutants. The soil surface itself acts as a buffer to temperature changes for the lower soil levels. Certain soil types such as clay can retain water for longer periods helping with soil moisture. With regard to the acidification process, it is a slow and natural process although it is hastened by pollution. Soil is the buffer against acidification because soil buffers external H^+^ ions, and only when the H^+^ input exceeds the maximum of soil buffer capacity, it will cause soil acidification [64]. Soil health can maintain the health of animals, plants, humans, and the environment [19], making it a vital system that needs to be monitored and protected. Soil pollutants such as heavy metals, organic contaminants, and persistent organic pollutants have been found in soil ecosystems and pose a threat to animal, human, and plant health [18]. The possibility of these pollutants affecting human and animal life is increasing with increasing pollution. Climate change effects are becoming more and more evident. The impact they have on soil pollutants is seen in ecosystems worldwide, making it a critical variable when performing ecotoxicological research. More research is needed to obtain adequate data on the interaction of environmental factors such as the temperature and moisture and other extreme climate change drivers. This has become more relevant with the acceleration of global climate and to enable relevant key climate regulatory authorities to implement laws to mitigate and manage the effects of the soil pollution on ecosystem health.

## Figures and Tables

**Table 1:** Effects of climate change parameters on the toxicity of pesticides towards earthworms.

Study	Climate change parameters					Earthworm species	Pesticide used	Effects on the life-cycle traits of earthworms and biomarkers
	Temperature	Precipitation/soil moisture	pH	Ultraviolet radiation	Acidification			
Friis et al. [76]	N/A	Soil water potentials from pF 1.5 (wet) to pF 5 (very dry) were obtained	N/A	N/A	N/A	*Aporrectodea caliginosa*	Copper	With the increasing drought level, the whole-body burden of copper increased from about 40 microg Cu g^−1^ dry weight to about 90 microg Cu g^−1^. When the worms were exposed to drought, the osmolality in their body fluids increased.
Booth et al. [77]	5–20°C	15–30% WHC	N/A	N/A	N/A	*Aporrectodea caliginosa*	N/A	ChE and GST activity increased with temperature.
Uvarov et al. [78]	Temperature fluctuations (2°C ± 5°C)	N/A	N/A	N/A	N/A	*Lumbricus rubellus* and *Dendrobaena octaedra*	N/A	Increase in temperature increased earthworm growth and reproduction rate.
Velki and Ečimović [79]	15, 20, and 25°C	N/A	N/A	N/A	N/A	*Eisenia fetida*	Imidacloprid, alpha-cypermethrin, indoxacarb, combined chlorpyrifos and cypermethrin, lambda-cyhalothrin, combined difenoconazole and propiconazole, combined azoxystrobin and cyproconazole, tembotrione, imazamox, diquat, fluazifop-p-butyl, glyphosate	Increase in temperature mostly resulted in increased pesticide toxicity, whereas toxicity decreased at lower temperature; investigation of mechanisms by which temperature affects the toxicity is required.
Lima et al. [80]	8, 20, and 28°C	55% WHC	5.8	N/A	N/A	*Eisenia andrei*	Carbaryl	Synergistic ratios showed a tendency to synergism at high temperatures; temperature increased the deleterious effects of carbaryl to *Eisenia andrei*.
De Silva et al. [81]	20 ± 2°C, 26 ± 2°C	50% WHC	5.9–6.1	N/A	N/A	*Eisenia andrei*	Carbendazim, carbofuran, and chlorpyrifos	Survival was more sensitive at the higher temperature; effects on reproduction and growth varied inconsistently with temperature and soil types; pesticide toxicity decreased in the order carbendazim > carbofuran > chlorpyrifos.
Bandeira et al. [82]	20, 25, and 28°C	60% WHC	6.0	N/A	N/A	*Eisenia andrei*	Imidacloprid	Toxicity of insecticides showed a tendency to increase with an increase in temperature, and this was evident both in the number of juveniles and the percentage of initial weight.
Zoua et al. [83]	20°C	35% WHC	Varied	N/A	Acidified soils (pH = 5.5, 4.3, and 3.1)	*Eisenia fetida*	Chlorpyrifos, triazophos, and dimethoate	The toxicity of OPs was slightly increased with the decrease of soil pH; bioavailability and toxicodynamics are key factors for toxicity variation.
Hackenberger et al. [84]	20 and 25°C	30% and 50% WHC	6.0 ± 0.5	N/A	N/A	*Eisenia fetida*	Propiconazole and chlorantraniliprole	Applied temperature and soil moisture combinations affected the selected biomarkers; the most important interaction was between concentration and temperature.

## Data Availability

The data used to support the findings of this study are available from the corresponding author upon request.
